# Prevalence and burden of headache in children and adolescents in Austria – a nationwide study in a representative sample of pupils aged 10–18 years

**DOI:** 10.1186/s10194-019-1050-8

**Published:** 2019-11-06

**Authors:** Julia Philipp, Michael Zeiler, Christian Wöber, Gudrun Wagner, Andreas F. K. Karwautz, Timothy J. Steiner, Çiçek Wöber-Bingöl

**Affiliations:** 1 Department of Child and Adolescent Psychiatry, Medical University of Vienna, Waehringer Guertel 18-20, 1090 Vienna, Austria; 2 Department of Neurology, Medical University of Vienna, Waehringer Guertel 18-20, 1090 Vienna, Austria; 3 Department of Neuromedicine and Movement Science, NTNU Norwegian University of Science and Technology, Trondheim, Norway; 4 Division of Brain Sciences, Imperial College London, London, UK; 5 Dr Gönül Bingöl-Dr Muammer Bingöl Çocuk ve Ergen Başağrısı Derneği, Society for Headache in Children and Adolescents, Tan Sokak 2/7, Suadiye, Istanbul, Turkey

**Keywords:** Epidemiology, Children and adolescents, Migraine, Tension-type headache, Medication-overuse headache, Undifferentiated headache, Quality of life, Global campaign against headache

## Abstract

**Background:**

Headache disorders are highly prevalent worldwide, but not so well investigated in children and adolescents as in adults: few studies have included representative nationwide samples. No data exist for Austria until now. In a representative sample of children and adolescents in Austria, we estimated the prevalence and attributable burden of headache disorders, including the new diagnostic category of “undifferentiated headache” (UdH) defined as mild headache lasting less than 1 hour.

**Methods:**

Within the context of a broader national mental health survey, children and adolescents aged 10–18 years were recruited from purposively selected schools. Mediated self-completed questionnaires included sociodemographic enquiry (gender, age, socioeconomic status, family constellation, residence [urban or rural] and migration background). Prevalence and attributable burden of all headache, UdH, migraine (definite plus probable), tension-type headache (TTH: definite plus probable) and headache on ≥15 days/month (H15+) were assessed using the Headache-Attributed Restriction, Disability, Social Handicap and Impaired Participation (HARDSHIP) questionnaire for children and adolescents. Health-related quality of life (HrQoL) was assessed using the KIDSCREEN questionnaire.

**Results:**

Of 7643 selected pupils, 3386 (44.3%) completed the questionnaires. The 1-year prevalence of headache was 75.7%, increasing with age and higher in girls (82.1%) than in boys (67.7%; *p* < 0.001). UdH, migraine, TTH and H15+ were reported by 26.1%, 24.2%, 21.6% and 3.0% of participants. Attributable burden was high, with 42% of those with headache experiencing restrictions in daily activities. Medication use (50% overall) was highest in H15+ (67%) and still considerable in UdH (29%). HrQoL was reduced for all headache types except UdH. Participants in single parent or patchwork families had a higher probability of migraine (respectively, OR 1.5, *p* < 0.001; OR 1.5, *p* < 0.01). Participants with a migration background had a lower probability of TTH (OR 0.7, *p* < 0.01).

**Conclusions:**

Headache disorders are both very common and highly burdensome in children and adolescents in Austria. This study contributes to the global atlas of headache disorders in these age groups, and corroborates and adds knowledge of the new yet common and important diagnostic category of UdH. The findings call for action in national and international health policies, and for further epidemiological research.

## Background

The Global Burden of Disease (GBD) study 2016 identified migraine as the second leading cause of years lived with disability (YLDs) [1], up from seventh in GBD2010 [2]. In adults, headache disorders are responsible for severe economic losses [3]. With published adult prevalences of 46–79% for any headache, 38–42% for tension-type headache (TTH), 11–35% for migraine, 3–7.2% for headache on ≥15 days/month (H15+) and 3.1% for probable medication-overuse headache (pMOH) [4, 5], these disorders are a major public-health problem worldwide, associated with impaired health-related quality of life (HrQoL) and high personal impact [5, 6].

In children and adolescents, headache disorders have the added and adverse potential of disrupting education [7], imposing a burden likely to be expressed throughout life [8]. In a Turkish/Austrian school-based sample, 21% of pupils had lost at least 1 day of school during the preceding 4 weeks because of headache, while 19% had left school early on at least 1 day for the same reason [7]. As in adults, HrQoL was poorer in pupils with headache than in those without [7, 9].

Although prevalence and attributable burden have frequently been investigated in these age groups (*eg*, [10–13]), there are few nationally representative epidemiological studies, and these are marked by methodological differences and leave large geographical gaps [14]. Two reviews have estimated the overall mean prevalence of headache in children and adolescents at 54.4–58.4%, with 7.7–9.1% migraine [10, 11]. In convenience-based school samples in Vienna and Istanbul, the 1-year prevalence of any headache was 89.3%, of migraine 39.3%, of TTH 37.9%, of H15+ 4.5% and of pMOH 0.8% [7]. In a later nationwide Turkish study, an important finding was that 37.2% of children and adolescents suffered from mild short-duration headache that could not be classified by standard criteria [14].

While a few earlier epidemiological studies had reported unclassifiable headaches, with an average prevalence of about 20% [12, 14, 15], most were silent on what appears to be a substantial proportion of affected children and adolescents. The Turkish study introduced a new diagnostic category of “undifferentiated headache” (UdH) [14], characterized by short duration (< 1 h) and mild intensity, the authors suggesting this was an immature form that would later evolve into migraine or TTH. Crucially, within an overall 1-year prevalence of headache of 73.7%, UdH was the most common category, diagnosed in 40% of the pupils with headache. The authors recommended inclusion of UdH in epidemiological studies not only to report the whole spectrum of headache disorders but also to give a full account of headache-attributed burden.

Headache in children tends to persist into adolescence and adulthood. Notably, 20–25% shifted from migraine to TTH or vice versa, a reflection, it would seem, of the same headache immaturity [11, 16]..

Altogether, headache is a highly prevalent and burdensome chronic recurrent disease in children and adolescents, with impacts on HrQoL, school attendance, social functioning and, predictably, later life [5, 8, 9]. Therefore, headache in these age groups is of substantial public-health importance. Epidemiological studies are essential to assess their prevalence, correlates and attributable burden, so that health and educational services can provide adequately for them [4, 8, 11, 17].

Until now, no such study exists for Austria. To redress this, and to contribute to the global atlas of headache disorders in children and adolescents, we performed this epidemiological study in a representative national sample of children and adolescents in Austria. It was an expansion of the *Mental Health in Austrian Teenagers* (MHAT) study [18, 19]. The questionnaires used in MHAT were supplemented with the Headache-Attributed Restriction, Disability, Social Handicap and Impaired Participation (HARDSHIP) questionnaire for children and adolescents.

This study assessed prevalence and burden of, and use of acute medication for, headache overall and each of the common specific headache disorders. Furthermore, it compared sociodemographic characteristics, and a broad spectrum of HrQoL domains, in participants without headache and those with the different headache disorders. Last but not least, it evaluated the necessity and sufficiency of the new diagnostic category of UdH in a second representative nationwide study.

## Methods

### Sample selection and recruitment

As recommended for epidemiological studies in children and adolescents [8], we used a cross-sectional design with a school-based sample. All 2547 secondary schools in Austria were asked to participate. Of those, 428 (16.8%) were willing to participate, with all school types in all Federal states in Austria were included among these. From those willing, a representative random sample of school classes was selected, stratified by grade (5th, 7th, 9th, 11th), school type and Federal state. Thus, the final selection of schools and school classes which were included in this study reflected the Austrian population of schools appropiately. All children and adolescents within these selected classes were included, except for those who refused to take part, were absent from school on the day of survey or were not competent in the German language. Data collection in each class was organized and mediated by a teacher. The questionnaires were administered during a school lesson (approximately 50 min) as online or paper-pencil versions. A pilot study earlier confirmed the feasibility and acceptability of these procedures [20]. More details are published elsewhere [21].

### Instruments

#### Headache and associated burden

The child and adolescent HARDSHIP questionnaire [7], as recommended by the Global Campaign against Headache [5, 8], consists of 44 questions including sociodemographic, screening and diagnostic questions and enquiries into burden in various domains and HrQoL. For this study, only the screening, diagnostic and burden questions were used. Diagnostic questions were based on the then current criteria of the International Classification of Headache Disorders (ICHD-3 beta) [22], which did not differ with respect to migraine and TTH from the now current ICHD-3 criteria. Burden questions referred to the numbers of days missed from school, leaving school early or with impaired everyday activities due to headache, within the previous four weeks. Additionally, we obtained self-reported school performance on a 4-point scale (1 = very good. 2 = good, 3 = average, 4 = below average). Data were obtained only from the children and adolescents themselves, since parents often underestimate the prevalence of headache and associated burden in their children [7].

#### Headache diagnoses

Diagnoses were derived using the HARDSHIP algorithm [5] applying ICHD-3 beta criteria [22] but with the recently published modifications to include UdH [14]. First, we applied the criteria for UdH (short duration [< 1 h] and mild intensity). From the remaining participants we separated those reporting H15+, and diagnosed pMOH when acute medication was used on ≥10 days/month, or otherwise “other H15+”. To participants reporting headache on < 15 days/month we applied diagnostic criteria, in order, for definite migraine, definite TTH, probable migraine and probable TTH. For TTH, we slightly modified criterion B (duration of headache) by raising the lower limit to 1 hour, as previously done by Wöber et al [14]. All participants with headache who fell into none of these categories were categorized as “unclassifiable headache”.

#### Sociodemographic variables

We enquired into gender, school grade, socioeconomic status (SES) of the family, migration background, family constellation and place of residence. To assess family SES, we used an extended version of the Family Affluence Scale [23], a self-report questionnaire for children and adolescents consisting of four items: number of cars in the family (none, one, two or more), having a room of one’s own (no, yes), frequency of holidays taken in the last 12 months (none, once, twice, more often), and number of computers within the family (none, one, two, more than two). We added two further items, used in the latest survey of *Health Behaviour in School-Aged Children Study* [24]: having a dishwasher (no, yes) and number of bathrooms (none, one, two, more than two). Item ratings were summed, with high scores indicating a high level of family affluence. SES was categorized as low (<25th percentile), medium (between 25th and 75th percentiles) and high (>75th percentile).

We recorded a migration background when either parent and/or the participating child or adolescent had been born in a foreign country. We categorized family constellation into three: living with biological parents, with a single parent or in a patchwork family. We categorized place of residence as rural (< 10,000 inhabitants) or urban (> 10,000 inhabitants).

#### Health-related quality of life

We used the KIDSCREEN questionnaire [25]: the KIDSCREEN-10 score as an overall measure of HrQoL, and additional domains of the KIDSCREEN-52 and KIDSCREEN-27 versions, including Self-Perception, Parent-Relations and Home Life, Social Support and Peers, School Environment, and Bullying (all Cronbach Alphas between .79 and .89). These items were rated on a 5-point scale; scores were translated into gender- and age-specific T-scores, with higher scores indicating higher HrQoL.

### Statistical analyses

We used IBM SPSS Statistics 25.0. Prevalence estimates (%) for any headache and for each headache type were calculated for the total sample and for each gender and school grade. We based standard errors (SEs) and confidence intervals (CIs) on simple random sampling: as there were a very high number of clusters (345 classes) and low numbers of participants within each cluster (n~ 10), the design effect was equal to 1 and, as a consequence, SEs based on cluster sampling were quite similar to SEs based on simple random sampling. We tested differences according to sociodemographic characteristics using chi-squared. We used logistic regression to analyze sociodemographic associations with each headache type including migraine (definite + probable), TTH (definite + probable), H15+ and UdH, calculating odds ratios (ORs); we used non-caseness (*ie*, all other headache types and no headache) as reference. We excluded unclassifiable headache from this analysis because of the low size of this group. In these regression models, all sociodemographic variables (gender, school grade, SES, family constellation, place of residence and migration background) were entered simultaneously, and only main effects were analyzed.

We analyzed impact of headache type on HrQoL, school attendance, school performance and everyday activities, as well as differences regarding medication use, using general linear models. For HrQoL, we additionally analyzed differences between participants with any headache and those with no headache using t-tests. We treated school-performance reports as continuous data, calculating means and SDs. We applied Bonferroni-adjusted significance values in view of the multiple comparisons of HrQoL measures and other measures of headache burden. We performed Tukey *post-hoc* tests for pairwise comparisons of headache types.

## Results

### Sample characteristics

After a stratified random sampling of classes (attended by a total of 7643 pupils), 3610 gave informed consent and 3470 completed the questionnaires. Of these, 84 were excluded from analyses because missing or inconsistent data prevented headache diagnosis. The final sample size was 3386 (participation proportion: 44.3%). Detailed descriptions of the sampling and flow of participants are published in Zeiler et al [21].

Sociodemographic characteristics of the sample are presented in Table 1.

**Table 1:** Headache prevalence by sociodemographic characteristics in the sample

	n (%)	Headache (any type) during the preceding year (%)		
		Yes	No	*p*
Total	3386 (100%)	75.7%	24.3%	
Gender				
Girls	1874 (55.4%)	82.1%	17.9%	Chi^2^ = 93.9, *p* < 0.001
Boys	1500 (44.5%)	67.7%	32.3%	
Missing	12			
School grade				
5th grade	496 (14.6%)	63.9%	36.1%	Chi^2^ = 49.9, *p* < 0.001
7th grade	865 (25.5%)	77.3%	22.7%	
9th grade	1087 (32.1%)	75.6%	24.4%	
11th grade	938 (27.7%)	80.4%	19.6%	
Socioeconomic status^a^				
Low	766 (23.4%)	76.2%	23.8%	Chi^2^ = 0.4, *p* = 0.808
Medium	1750 (53.5%)	76.1%	23.9%	
High	756 (23.1%)	75.0%	25.0%	
Missing	114			
Family constellation				
Both biological parents	2437 (74.3%)	74.8%	25.2%	Chi^2^ = 7.2, *p* = 0.027
Single parent	547 (16.7%)	78.8%	21.2%	
Patchwork	295 (9.0%)	79.7%	20.3%	
Missing	107			
Place of residence^a^				
Urban	1955 (58.5%)	75.9%	24.1%	Chi^2^ = 0.4, *p* = 0.524
Rural	1385 (41.5%)	74.9%	25.1%	
Missing	46			
Migration background^a^				
No	2470 (74.3%)	76.4%	23.6%	Chi^2^ = 2.9, *p* = 0.088
Yes	855 (25.7%)	73.5%	26.5%	
Missing	61			

^a^See text for explanations

### Prevalence of headache

The 1-year prevalence of all headache in the entire sample was 75.7% [95% CI: 74.3; 77.1], higher in girls (82.1%) than in boys (67.7%) (Table 1). Overall, the most common diagnosis was UdH (26.1%; 26.5% in girls, 25.5% in boys; 34.5% of those with any headache). This was followed by migraine (24.2%; 28.1% in girls, 19.5% in boys; 32.6% of those with headache) and TTH (21.6%; 22.4% in girls, 20.7% in boys; 28.5% of those with headache) (Table 2). All H15+ was diagnosed in 3.0% (4.1% in girls, 1.7% in boys; 3.9% of those with any headache) and pMOH in 0.9% (1.0% in girls, 0.9% in boys). Thus, only migraine and other H15+ were marked by female predisposition (Table 2). Prevalence of headache increased with age from 63.9% in 5th grade to 80.4% in 11th grade (*p* < 0.001) (Table 1). The drivers were migraine in girls (increasing from 18.1% in 5th grade to 32.5% in 11th grade) and TTH in both genders (in girls from 14.3% to 25.6% and in boys from 15.6% to 27.4%), although the overall increase was partially offset by a decrease in UdH in girls (from 32.8% to 23.3%) but not boys (Table 2). Overall, UdH declined as a proportion of all headache with increasing maturity, in favour of the more specific headache types, from 44.2% in 5th grade to 29.2% in 11th grade (*p* < 0.05) (Table 3).

**Table 2:** Prevalence (% [95% confidence interval]) of headache type (*N* = 3386) by gender and school grade

Headache type	Total sample	Girls					Boys				
		Total	5th grade	7th grade	9th grade	11th grade	Total	5th grade	7th grade	9th grade	11th grade
UdH	26.1 [24.6;27.6]	26.5 [24.5;28.5]	32.8 [26.8;38.8]	31.5 [27.1;35.9]	23.7 [20.4;27.0]	23.3 [19.8;26.8]	25.5 [23.3;27.7]	23.8 [18.6;29.0]	31.2 [26.8;35.6]	22.4 [18.5;26.3]	23.7 [19.4;28.0]
All migraine	24.2 [22.8;25.6]	28.1 [26.1;30.1]	18.1 [13.2;23.0]	23.1 [19.1;27.1]	31.4 [27.8;35.0]	32.5 [28.6;36.4]	19.5 [17.5;21.5]	17.2 [12.6;21.8]	21.2 [17.3;25.1]	18.8 [15.2;22.4]	19.9 [15.8;24.0]
Definite migraine	6.3 [5.5;7.1]	6.9 [5.8;8.0]	10.1 [6.3;13.9]	5.3 [3.2;7.4]	5.3 [3.6;7.0]	8.7 [6.4;11.0]	5.5 [4.3;6.7]	10.9 [7.1;14.7]	4.7 [2.7;6.7]	4.1 [2.7;6.7]	4.3 [2.2;6.4]
Probable migraine	18.0 [16.7;19.3]	21.2 [19.3;23.1]	8.0 [4.5;11.5]	17.8 [14.2;21.4]	26.1 [22.7;29.5]	23.8 [20.3;27.3]	14.0 [12.2;15.8]	6.3 [3.3;9.3]	16.5 [13.0;20.0]	14.7 [11.4;18.0]	15.6 [11.9;19.3]
All TTH	21.6 [20.2;23.0]	22.4 [20.5;24.3]	14.3 [9.8;18.8]	23.1 [19.1;27.1]	22.0 [18.8;25.2]	25.6 [22.0;29.2]	20.7 [18.6;22.8]	15.6 [11.6;20.1]	18.1 [14.5;21.7]	20.4 [16.6;24.2]	27.4 [22.9;31.9]
Definite TTH	15.1 [13.9;16.3]	15.2 [13.6;16.8]	8.4 [4.9;11.9]	14.4 [11.1;17.7]	15.0 [12.2;17.8]	19.0 [15.8;22.2]	14.9 [13.1;16.7]	12.9 [8.8;17.0]	13.7 [10.4;17.0]	13.1 [10.0;16.2]	19.9 [15.8;24.0]
Probable TTH	6.5 [5.7;7.3]	7.2 [6.0;8.4]	5.9 [2.9;8.9]	8.8 [6.1;11.5]	7.0 [5.0;9.0]	6.6 [4.5;8.7]	5.7 [4.5;6.9]	2.7 [0.7;4.7]	4.4 [2.5;6.3]	7.2 [4.8;9.6]	7.5 [4.8;10.2]
All H15+	3.0 [2.4;3.6]	4.1 [3.2;5.0]	4.2 [1.6;6.8]	3.0 [1.4;4.6]	5.5 [3.7;7.3]	3.2 [1.7;4.7]	1.7 [1.0;2.4]	0.8 [0.0;1.9]	1.4 [0.3;2.5]	2.3 [0.9;3.7]	1.9 [0.5;3.3]
pMOH	0.9 [0.6;1.2]	1.0 [0.5;1.5]	0.4 [0.0;1.2]	0.5 [0.0;1.2]	1.2 [0.4;2.0]	1.4 [0.4;2.4]	0.9 [0.4;1.4]	0.8 [0.0;1.9]	0.5 [0.0;1.2]	1.4 [0.3;2.5]	0.8 [0.0;1.7]
Other H15+	2.0 [1.5;2.5]	3.0 [2.2;3.8]	3.8 [1.4;6.2]	2.5 [1.0;4.0]	4.2 [2.6;5.8]	1.8 [0.7;2.9]	0.8 [0.3;1.3]	0.0	0.9 [0.0;1.8]	0.9 [0.0;1.8]	1.1 [0.0;2.2]
Unclassifiable	0.7 [0.4;1.0]	1.0 [0.5;1.5]	1.3 [0.0;2.7]	1.6 [0.4;2.8]	0.8 [0.1;1.5]	0.7 [0.0;1.4]	0.4 [0.1;0.7]	0.4 [0.0;1.2]	0.5 [0.0;1.2]	0.5 [0.0;1.2]	0.3 [0.0;0.9]

*UdH* Undifferentiated headache, *TTH* Tension-type headache, *H15+* Headache on ≥15 days/month, *pMOH* Probable medication-overuse headache (see text)

**Table 3:** Headache diagnoses by school grade among pupils (both genders) reporting headache during the preceding year

Headache type	Proportions of those with any headache (*N* = 2562)			
	5th grade (*n* = 317)	7th grade (*n* = 669)	9th grade (*n* = 822)	11th grade (*n* = 754)
UdH	44.2%^a^	40.5%^a^	30.8%^b^	29.2%^b^
All migraine	27.4%^a^	28.5%^a^	34.6%^a^	34.2%^a^
Definite migraine	16.4%^a^	6.4%^b^	6.3%^b^	8.6%^b^
Probable migraine	11.0%^a^	22.1%^b^	28.3%^c^	25.6%^b,c^
All TTH	23.3%^a^	26.7%^a,b^	28.3%^a,b^	32.6%^b^
Definite TTH	16.7%^a,b^	18.2%^b^	18.9%^a,b^	24.0%^a^
Probable TTH	6.6%^a^	8.5%^a^	9.4%^a^	8.6%^a^
All H15+	3.7%^a^	2.8%^a^	5.5%^a^	3.4%^a^
pMOH	0.9%^a^	0.6%^a^	1.7%^a^	1.5%^a^
Other H15+	2.8%^a^	2.2%^a^	3.8%^a^	1.9%^a^
Unclassifiable	1.3%^a^	1.3%^a^	0.9%^a^	0.7%^a^

*UdH* Undifferentiated headache, *TTH* Tension-type headache, *H15+* Headache on ≥15 days/month, *pMOH* probable medication-overuse headache (see text); ^a,b,c^ These superscript notations indicate groups differing (*p* < 0.05) on Tukey *post-hoc* analyses

### Sociodemographic correlates

Gender, school grade and family constellation differed between participants with and without headache, whereas SES, place of residence and migration background did not (Table 1).

However, some of these sociodemographic characteristics were more associated with specific headache types. With regard to overall model fit, the sociodemographic characteristics included in the logistic regression models significantly predicted headache type (in all cases, *p* < 0.001), whereas the explained variance was low (Nagelkerke *R*^*2*^ = 0.018 to 0.037). Female gender was associated with migraine (OR 1.5) and H15+ (OR 2.4). Higher school grades were associated with migraine and TTH; older participants had a higher probability of these headache types. With regard to family constellation, participants living in single parent or patchwork families had a higher probability of migraine (OR 1.5) than those living with both biological parents. Participants with a migration background showed a lower probability of TTH (OR 0.7). Table 4 provides intercepts and slopes of the regression models as well as ORs with 95% CIs for these associations.

**Table 4:** Logistic regression analyses of sociodemographic associations with headache type

	b (SE)	95% CI for Odds Ratio		
		Lower	OR	Upper
UdH vs no UdH (Nagelkerke *R*^*2*^ = .018; Model Chi^2^ = 37.79; *p* < 0.001)				
Intercept	−1.08 (0.15)***			
Female gender (ref male)	0.05 (0.08)	0.9	1.1	1.2
School grade (ref 5th grade)				
7th grade	0.17 (0.13)	0.9	1.2	1.5
9th grade	−0.22 (0.13)	0.6	0.8	1.0
11th grade	−0.26 (0.14)	0.6	0.8	1.0
Socioeconomic status (ref high)				
Medium	0.12 (0.11)	0.9	1.1	1.4
Low	0.26 (0.13)*	1.0	1.3	1.7
Family constellation (ref both parents)				
Single parent	−0.24 (0.12)*	0.6	0.8	1.0
Patchwork	−0.33 (0.16)*	0.5	0.7	1.0
Urban place of residence (ref rural)	−0.05 (0.09)	0.8	1.0	1.1
Migration background (ref no)	0.21 (0.10)*	1.0	1.2	1.5
Migraine vs no migraine (Nagelkerke *R*^*2*^ = .031; Model Chi^2^ = 63.69; *p* < 0.001)				
Intercept	−1.87 (0.16)***			
Female gender (ref male)	0.39 (0.09)***	1.2	1.5	1.8
School grade (ref 5th grade)				
7th grade	0.32 (0.15)*	1.0	1.4	1.8
9th grade	0.49 (0.15)**	1.2	1.6	2.2
11th grade	0.52 (0.15)***	1.3	1.7	2.2
Socioeconomic status (ref high)				
Medium	−0.07 (0.11)	0.8	0.9	1.1
Low	−0.11 (0.13)	0.7	0.9	1.2
Family constellation (ref both parents)				
Single parent	0.41 (0.11)***	1.2	1.5	1.9
Patchwork	0.40 (0.14)**	1.1	1.5	2.0
Urban place of residence (ref rural)	0.12 (0.09)	0.9	1.1	1.3
Migration background (ref no)	0.01 (0.10)	0.8	1.0	1.2
TTH vs no TTH (Nagelkerke *R*^*2*^ = .023; Model Chi^2^ = 45.50; *p* < 0.001)				
Intercept	−1.56 (0.17)***			
Female gender (ref male)	0.12 (0.10)	0.9	1.1	1.4
School grade (ref 5th grade)				
7th grade	0.37 (0.16)*	1.1	1.4	2.0
9th grade	0.43 (0.16)**	1.1	1.5	2.1
11th grade	0.73 (0.16)***	1.5	2.1	2.8
Socioeconomic status (ref high)				
Medium	−0.11 (0.11)	0.7	0.9	1.1
Low	−0.16 (0.14)	0.7	0.9	1.1
Family constellation (ref both parents)				
Single parent	−0.02 (0.12)	0.8	1.0	1.3
Patchwork	0.02 (0.16)	0.8	1.0	1.4
Urban place of residence (ref rural)	−0.12 (0.09)	0.7	0.9	1.1
Migration background (ref no)	−0.34 (0.11)**	0.6	0.7	0.9
H15+ vs. no H15+ (Nagelkerke *R*^*2*^ = .037; Model Chi^2^ = 26.59; *p* = 0.003)				
Intercept	−3.97 (0.41)***			
Female gender (ref male)	0.86 (0.25)**	1.4	2.4	3.8
School grade (ref 5th grade)				
7th grade	−0.29 (0.38)	0.4	0.7	1.6
9th grade	0.23 (0.34)	0.7	1.3	2.5
11th grade	−0.19 (0.37)	0.4	0.8	1.7
Socioeconomic status (ref high)				
Medium	−0.13 (0.26)	0.5	0.9	1.5
Low	−0.52 (0.35)	0.3	0.6	1.2
Family constellation (ref both parents)				
Single parent	0.40 (0.28)	0.9	1.5	2.6
Patchwork	0.60 (0.32)	1.0	1.8	3.4
Urban place of residence (ref rural)	0.01 (0.22)	0.7	1.0	1.6
Migration background (ref no)	−0.24 (0.27)	0.5	0.8	1.4

*UdH* Undifferentiated headache, *TTH* Tension-type headache, *H15+* Headache on ≥15 days/month; *p* < 0.001, * *p* < 0.05, **.*p* < 0.01, *** *p* < 0.001

### Impact of headache on education

During the preceding 4 weeks, 15.6% of participants with headache missed at least one whole school day because of headache, while 11.7% left school early at least once; 41.9% reported at least 1 day on which they were unable to do other activities they had wanted to. These proportions varied with headache type, being highest in those with H15+, next highest in those with migraine and lower in TTH and UdH (Table 5). Nevertheless, reported school performance was similar in all diagnostic groups, with a tendency to be poorer in those with H15+ or migraine than in those with TTH, UdH or no headache (Table 5).

**Table 5:** Burden of headache, acute medication use and health-related quality of life by headache diagnosis (*N* = 2562)

	No headache (*n* = 824)	UdH (*n* = 884)	All migraine (*n* = 821)	All TTH (*n* = 731)	H15+ (*n* = 101)	Test statistic, *p*-value^f^
Headache burden (reported at least once in previous 4 weeks) (% of participants)						
Headache-attributed lost time:						
Missed whole school day	–	11.0%^a^	20.5%^b^	11.3%^a^	31.3%^c^	Chi^2^ = 47.3, *p* < 0.001
Left school early	–	7.6%^a^	15.2%^b^	9.0%^a^	24.2%^c^	Chi^2^ = 35.6, *p* < 0.001
Missed other activities	–	26.8%^a^	57.3%^b^	34.5%^c^	60.2%^b^	Chi^2^ = 141.6, *p* < 0.001
School performance (mean, SD)^e^	2.1 (0.8)^a, b^	2.1 (0.8)^a^	2.3 (0.8)^b^	2.2 (0.8)^a, b^	2.3 (0.90)^b^	*F* = 6.7, *p* < 0.001
Acute medication use in previous 4 weeks						
Participants (%)	–	28.5^a^	58.7^b^	43.9^c^	67.3^b^	Chi^2^ = 128.9, *p* < 0.001
Number of days (mean, SD)	–	0.8 (2.5)^a^	1.8 (2.6)^b^	1.0 (1.7)^a^	6.5 (7.6)^c^	*F* = 121.4, *p* < 0.001
Health-related quality of life (HrQoL)						
Overall HrQoL	52.9 (10.8)^a^	53.9 (9.5)^a^	47.5 (11.2)^b^	50.6 (10.4)^c^	43.1 (11.9)^d^	*F* = 56.3, *p* < 0.001
Self-perception	51.0 (9.7)^a^	50.9 (9.2)^a^	46.4 (10.0)^b^	48.2 (9.9)^b^	42.9 (10.8)^c^	*F* = 42.3, *p* < 0.001
Parent-relation and home life	53.9 (9.2)^a^	53.6 (8.7)^a^	50.3 (11.1)^b^	51.9 (9.7)^a, b^	44.8 (13.9)^c^	*F* = 31.6, *p* < 0.001
Social support and peers	52.6 (9.4)^a^	53.1 (8.7)^a^	51.6 (9.8)^a^	52.1 (8.8)^a^	49.6 (13.2)^b^	*F* = 4.8, *p* = 0.001
School environment	52.7 (10.5)^a, c^	53.4 (8.8)^a^	48.4 (10.1)^b^	50.9 (9.3)^c^	45.6 (12.6)^d^	*F* = 39.5, *p* < 0.001
Bullying	50.7 (10.6)^a^	51.9 (8.9)^a^	50.4 (10.2)^a^	50.7 (9.7)^a^	47.3 (14.3)^b^	*F* = 6.4, *p* < 0.001

*UdH* Undifferentiated headache, *TTH* Tension-type headache, *H15+* Headache on ≥15 days/month. ^a,b,c,d^These superscript notations indicate groups differing significantly on Tukey *post-hoc* analyses. ^e^On scale 1–4 where 1 = very good, 4 = below average (see text). ^f^Bonferroni corrections applied to significance values to adjust for multiple comparisons of headache burden measures (*p* = 0.013) and of HrQoL measures (*p* = 0.008)

### Use of acute headache medication

Among those with headache, 49.6% reported intake of acute (abortive) headache medication during the preceding 4 weeks, with, on average, medication use on 1.5 days. Frequency of use was much higher in H15+, but still only on a mean of 6.5 days (Table 5).

### Health-related quality of life

HrQoL scores were reduced in participants with any headache compared with those with no headache on overall KIDSCREEN-10 score and on scores for self-perception, parent-relations and home life, and school environment (all *p* < 0.001). There were no differences in social support and peers (*p* = 0.310) or bullying (*p* = 0.626). Looking at different headache types specifically, we observed significant differences in HrQoL for all KIDSCREEN domains. In all these domains, there was a gradient: H15+ < migraine < TTH < UdH, with no differences in any domain between UdH and no headache (Table 5).

## Discussion

This first representative survey in Austria obtained data on headache from a large sample of 3386 children and adolescents aged 10–18 years. More than 75% had experienced headache in the previous year. The overall prevalences of migraine and TTH were 24.2% and 21.6% respectively, while 3.0% of participants reported H15+, including 0.9% with pMOH. Importantly, 26.1% had headache classified as UdH, corroborating the earlier findings in Turkey [14]. More than 40% of participants with headache reported at least 1 day of lost activity during the previous 4 weeks, and almost 50% used acute medication on at least 1 day. On average, medication was used on 1.5 days. While it was much higher in H15+, the mean of 6.5 days was notably fewer than headache days (by definition, ≥15). These age groups do not always need or benefit from medication.

While UdH was the most common headache type, its prevalence declined with increasing age. At the same time, the proportions with migraine and TTH increased, supporting the proposal of Wöber et al that UdH is a precursor or immature form of these headache types [14] (or, perhaps more accurately, expressions of these headache types by an immature brain). Only longitudinal studies can prove this. Meanwhile it should be noted that, although UdH is mild and of relatively short duration, more than one quarter of participants with UdH reported at least 1 day of lost activity as well as use of acute medication during the previous 4 weeks. The quite high medication use in UdH demonstrates the need to educate both, the affected children and adolescents as well as the parents about the effects and latency of analgesic drugs. It is important to recommend profound diagnostic assessment in a specialist centre and to propose alternative treatment strategies (i.e. relaxation techniques) when appropriate to prevent medication overuse.

In this study, headache prevalence was higher than reported in earlier reviews [10, 11], but comparable to those from a nationwide study in Turkey also applying the Child and Adolescent HARDSHIP questionnaire (bearing in mind that the Turkish study included 6–18-year-olds) [14]. The discrepancy against earlier studies can be explained by crucial differences in methodology in these studies, which, especially, have tended selectively to report definite but not probable migraine and TTH, exclude mild headaches and ignore undifferentiated or unclassifiable headaches [14]. The prevalence of UdH here was quite similar to that found in Turkey, in the only study so far to have reported it (again bearing in mind that this study included 6–18-year-olds) [14].

Children and adolescents reported substantial impact of headache on health and daily life. HrQoL was poorer in those with headache, overall and in most domains, with, in accordance with the literature [4, 6, 7, 9, 14], a general gradient: poorest in H15+, then migraine, then TTH. Crucially, headache had an adverse effect on educational attendance – potentially creating a cumulative, lifelong burden [5]: while > 40% lost at least 1 day of an activity they liked to do, 16% lost at least 1 day of school and 12% had to leave school early at least once within the preceding 4 weeks. As expected [26, 27], these impacts were greatest for H15+, followed by migraine, TTH and UdH, results similar to those from Turkey [14]. Although the proportions reporting missed daily activities, school days or lessons were lowest in UdH compared with other headache types, they were still noteworthy (27%, 11%, 8%), clear evidence that the burden of headache is substantially underestimated if UdH is not included.

Female gender, higher school grade and living in single parent or patchwork families were all factors in our study associated with increased probability of headache. Girls have consistently been reported to suffer more than boys from any headache and from migraine [10, 11, 28]. We found female gender to be associated with higher prevalences of any headache, of migraine and of H15+. Among children and adolescents, headache prevalence has, with similar consistency, been reported to increase with age [10, 12, 28, 29]. We found the same, overall and for all headache types except for H15+ and, of course, UdH. Children and adolescents with divorced parents and those living with a single parent have earlier reported higher prevalences of headache [9, 30, 31]. We found family constellation similarly to be a factor in the prevalences of any headache and of migraine.

Less consistent have been the findings regarding SES, place of residence or migration. Some studies have found no relation between headache in general and SES [9, 10, 31], with some exceptions in specific subgroups (*eg*, preschool children) [9]. Another has described an association between migraine and SES, but only in families without a family history of migraine [32]. We agreed with the first [9, 10, 31], finding no clear relationship between SES and any headache type. We also agreed with these studies [9, 10, 31], but not with others [28, 32, 33], that urban vs rural place of residence did not differ between those with and those without headache. Information about migration and headache is limited. One study showed a correlation between migration background of the family and prevalence of headache in children [33]. We found only that a migration background was associated with a lower probability of TTH, in an analysis possibly biased by our exclusion of non-German speakers, leading probably to underrepresentation of recent migrants.

Our study had important limitations. Questionnaires, as always in such studies, introduce recall problems and possible misunderstandings. As this study was part of the broader MHAT study, and the data collection time was limited to one school lesson, enquiry about headache yesterday and further impact questions in the HARDSHIP questionnaire could not be included. Because of the cross-sectional design of the study, associations between headache and age, gender, other sociodemographic variables and HrQoL could only be noted, and not interpreted in terms of causation. The most serious limitation was the high non-participation proportion: at 55.7%, it was above the limit of 50% deemed acceptable [8]. While absences on the day of data collection (possibly for headache) might have contributed to a small extent, studies in these age groups are rendered very difficult when, as here, prior written parental (or guardian) consent is required. The problem is passive non-response rather than active objection, and there is no obvious solution to it [8]. Generally, non-participation increases risk of overestimation due to interest bias (pupils without headache are less willing to take part) [8], but this might not have been the case here because the MHAT study (and, therefore, interest) was not focused on headache. Furthermore, a non-responder analysis reported elsewhere indicated only a small risk of bias [34]. Another limitation might be the included age group of students of 10–18 years. Headache with a short duration is known to be especially prevalent in children younger than 6 years [35]. As UdH is expected to be an immature form of headache with a higher prevalence in younger children, the prevalence of UdH might increase with the inclusion of younger children.

The study had strengths also. It generated a large random stratified sample representative of the whole country, unlike most published studies in children and adolescents. Also in contrast to almost all previous studies, it accounted for all reported headaches, being only the second study to address UdH [14]. Thus, this study makes a contribution to the aim of the Global Campaign against Headache to close the gap of missing evidence concerning headache and its attributable burden worldwide, especially, more recently, in children and adolescents [4, 8, 17]. It followed the Campaign’s consensus recommendations for high-quality epidemiological research on headache [8], applying a widely-used questionnaire and making headache diagnoses according to ICHD-3 beta [22] and through a standard algorithm to facilitate comparisons between countries [5, 8].

Upcoming research should address UdH as probable immature form of other headache types as well as associated burden and mental health problems in adolescents, which is also of national and international significance [36, 37].

## Conclusion

Headache disorders are very common in children and adolescents in Austria, as they are in other countries worldwide. They are burdensome in terms of ill health, impaired quality of life and interference with education. While the general impact gradient declines from H15+ through migraine and TTH to UdH, burden and medication use are far from negligible even in the last. This study confirms that UdH, a new diagnostic category, is very common in children and adolescents, while supporting the hypothesis that UdH may be a precursor or immature form of migraine or TTH. UdH needs careful attention in future epidemiological studies.

Our results contribute to the global atlas of headache disorders in children and adolescents, and reconfirm that headache disorders are highly relevant to health policy.

## Data Availability

The data that support the findings and conclusions of this study article are located electronically at the Department of Child and Adolescent Psychiatry, Medical University of Vienna, Vienna, Austria. They are not yet publicly available as they are still being analyzed, with some further publications expected.

## Abbreviations

H15+: Headache on ≥15 days/month
HARDSHIP: Headache-Attributed Restriction, Disability, Social Handicap and Impaired Participation
HrQoL: Health related quality of life
ICHD: International Classification of Headache Disorders
MHAT: Mental Health in Austrian Teenagers
pMOH: probable medication-overuse headache
TTH: Tension-type headache
UdH: Undifferentiated headache
